# Chemical Similarity Enrichment Analysis (ChemRICH) as alternative to biochemical pathway mapping for metabolomic datasets

**DOI:** 10.1038/s41598-017-15231-w

**Published:** 2017-11-06

**Authors:** Dinesh Kumar Barupal, Oliver Fiehn

**Affiliations:** NIH-West Coast Metabolomics Center, Genome Center University of California, Davis, USA

## Abstract

Metabolomics answers a fundamental question in biology: How does metabolism respond to genetic, environmental or phenotypic perturbations? Combining several metabolomics assays can yield datasets for more than 800 structurally identified metabolites. However, biological interpretations of metabolic regulation in these datasets are hindered by inherent limits of pathway enrichment statistics. We have developed ChemRICH, a statistical enrichment approach that is based on chemical similarity rather than sparse biochemical knowledge annotations. ChemRICH utilizes structure similarity and chemical ontologies to map all known metabolites and name metabolic modules. Unlike pathway mapping, this strategy yields study-specific, non-overlapping sets of all identified metabolites. Subsequent enrichment statistics is superior to pathway enrichments because ChemRICH sets have a self-contained size where *p*-values do not rely on the size of a background database. We demonstrate ChemRICH’s efficiency on a public metabolomics data set discerning the development of type 1 diabetes in a non-obese diabetic mouse model. ChemRICH is available at www.chemrich.fiehnlab.ucdavis.edu

## Introduction

Much remains to be learned about metabolism^1–4^. Metabolism produces energy and building blocks which are used for reproduction^5^, protection^6^, communication^7^, maintenance^8^ and structure^9^ of cells. Diseases, genetic and environmental factors interact with quantitative and qualitative changes in metabolism^10–12^. Understanding those changes and their biological effects on organism’s life is one of the basic questions of biology with importance ranging from synthetic biology to understanding the onset and progress of chronic diseases^13^. Over the past two decades, metabolomics technologies have been developed to investigate these metabolic changes on a comprehensive scale^14–16^.

Today, metabolomics datasets often contain 500–800 structurally identified compounds, for example in biomedical research^17–23^, including tumor tissues^24^. Metabolomics workflows aim to measure a maximum number of metabolites in a specific biological situation with as few technology platforms as possible^25,21,19,26^. Using online statistical tools^27^ and standardized and open-source R scripts^28^, it is straightforward to perform a range of statistical analyses for these datasets. For many studies, statistical significance values and effect sizes are publicly available for all individual metabolites in addition to multivariate metabolic phenotypes^29^

However, a major challenge in metabolomics is the biological interpretation of the observed metabolic changes. A first step is to combine observed changes into categories.

In genomics, set enrichment analysis is a major step in the interpretation of results^30^. It is a statistical procedure in which a *p*-value for a set of pre-defined variables is obtained to indicate that a set is enriched with respect to the input list of variables. Commonly used set definitions are pathway maps^31^, ontology terms^32^ and other functional sets^33^. Pathway maps are static layouts of metabolic reactions and are often used as set definitions for metabolite over-representation analysis^28,34–36^. Maps provided by KEGG^31^, SMPDB^37^, reactome^38^ and BioCyc^39^ databases define pathways by human curators, but their organization follows different logics and are often hard to compare to each other. Moreover, metabolomics assays miss many pathway intermediates, either because of low abundance or because of chemical and biological constraints. Third, for many identified compounds, especially lipids, substrate-enzyme relationships have not been unambiguously defined^40^. Fourth, many metabolites appear in multiple pathways or pathway maps even when using just a single database. Hence, these compounds would contribute to significance testing in multiple times, distorting biological interpretations.

Instead of using biochemical pathway annotations for mapping metabolites into separate sets of molecules, a more logical way is to use molecule’s chemical structure itself. Classically, chemical structures have been named by groups such as “sugars”, or “amino acids”. The problem for such annotations lies in the chemical diversity of metabolomes that transcend such straightforward classification. For example, glycolipids have sugar-like moieties as well as fatty acyl chains, argininosuccinate consists of an amino acid and a dicarboxylic acid. Nevertheless, efforts for classifications of chemicals have progressed tremendously over the past 60 years. Chemical ontologies in the Medial Subject Headings (MeSH) database have been established to classify 16,000 groups of chemicals to a much greater detail than other tools such as ClassyFire or ChEBI. However, even MeSH ontology term annotations alone are not sufficient for metabolomics. Especially lipids are sparsely covered by MeSH. Moreover, often more than one term is mapped to a single metabolite, requiring set level *p*-values to be corrected for false discovery rates as more overlapping sets and terms are included in the enrichment analysis. We here propose using MeSH annotations and Tanimoto chemical similarity calculations to define sets of related molecules in metabolomics assays in a unique and non-overlapping way.

Once such variable sets have been defined, significance *p*-values can be calculated using a range of statistical tests. Selecting an appropriate test depends on two major criteria: the background database and the input list of variables. In gene expression analysis, set enrichment is well established^41^. Here, the background database could be either all the assayed genes or all genes in a genome^41^. The input gene list can then include all differentially regulated genes from univariate statistics, or otherwise selected lists (e.g. most-important-variables from multivariate statistics, genes resulting from regression analyses or other statistical outputs). Next, the actual set-specific significance levels are calculated, and here, differences become apparent between genomics and metabolomics. Two different tests are used. In metabolomics, the hypergeometric test or the Fisher exact test are used very often^34^. The problem with both tests is that significance levels entirely depend on the size of background database to calculate the *p*-values. A large sized database such as PubChem with 70 million entries gives much lower *p*-values than using a small-sized database such as the KEGG Ligand database with only 23,617 entries. These tests assume that the background database is static, as it is for genomics. This assumption is false because the size of the metabolome is not static and today may extend into exposome analyses^13^. If one would simply use all the assayed variables as study-specific background database for a hypergeometric test, p-values would differ from assay to assay, depending on the number of metabolites detected. However, the number of potential chemicals detected in an untargeted metabolomics screen is not static. An alternative for enrichment statistics is presented by the Kolmogorov–Smirnov (KS) test^42^ and binomial tests. Both tests are self-contained. Their significance *p*-values do not rely on the size of background databases^41^. We propose using the KS algorithm because it tests for the distribution of *p*-values and, hence, does not require a hard *p*-value cut-off threshold.

Here we present ChemRICH, a chemical similarity enrichment analysis software for metabolomics datasets. It utilizes medical subject headings and Tanimoto substructure chemical similarity coefficients to cluster metabolites into non-overlapping chemical groups. On these clusters, statistical significance *p*-values are obtained by self-contained Kolmogorov–Smirnov tests. We showcase the suitability of this approach on a published case study that compared the serum metabolome of non-obese diabetic mice versus control animals. The tool is provided as an R-package and as an online web-app.

## Material and Methods

### Metabolomics dataset

Data were downloaded from the NIH MetabolomicsWorkbench.org database^29^ as study number ST000075. Compound identifiers and SMILES code metabolite were obtained from the Chemical Translation Service (CTS) (http://cts.fiehnlab.ucdavis.edu/) and PubChem identifier exchange service (https://pubchem.ncbi.nlm.nih.gov/idexchange).

### Pathway mapping using the NCBI BioSystems database

Metabolites were linked to conserved biological pathways obtained from NCBI BioSystem database ftp location (ftp://ftp.ncbi.nih.gov/pub/biosystems)^43^. PubChem identifiers of metabolites were queried against the NCBI BioSystems database as single point access across five databases, namely KEGG, BioCyc, Reactome, GO and Wikipathways.

### Medical Subject Headings (MeSH) database

The MeSH ontology files (https://www.nlm.nih.gov/mesh/download_mesh.html) were downloaded both the main and supplement terms and processed in R to make a table of entry metabolite terms, MeSH tree identifiers (e.g. D03.633.100.473) and MeSH term identifier (e.g. 68007211).

### Mapping PubChem identifiers to MeSH terms

Compounds were mapped to MeSH terms from the NCBI resource (ftp://ftp.ncbi.nih.gov/pubchem/Compound/Extras/CID-MeSH). This file contains around 110,000 PubChem compound identifiers (CID) corresponding to around 85,000 unique compounds. The number of CIDs annotated for each MeSH term categories was obtained from the PubChem database (https://pubchem.ncbi.nlm.nih.gov/classification/). We retrieved MeSH tree identifiers for each CID by lookups for each MeSH term that at least one PubChem CID. The resulting file had 114,954 entries is provided as an R data object in the ChemRICH R-package.

### Processing lipid compounds

String patterns for identifying fatty acids compounds including saturated, unsaturated, hydroxyl, epoxy and oxy fatty acids and prostaglandins were coded in R. For each compound the SMILES code was queried for these string patterns using regular expression search in R. SMILES for lipid compounds were searched for “C=C” pattern to detect the presence of double bonds. Classes of lipids were annotated as unsaturated lipids if at least three compounds in that class contained double bonds.

### Calculation of PubChem substructure fingerprint

SMILES codes were converted into an rCDK molecule container which was then used to calculate the PubChem 881 bit fingerprint (ftp://ftp.ncbi.nlm.nih.gov/pubchem/specifications/pubchem_fingerprints.txt). Fingerprints for representative compounds for each lipid class and selected MeSH classes were calculated and stored in an R object for re-use.

### Storing ChemRICH sets in a database

A NoSQL database Apache CouchDB version 1.6.0 was used for storing the MeSH annotations and the substructure fingerprints for metabolites to enable fast queries.

### Calculation of pair-wise chemical similarity matrices

Using the PubChem 881 fingerprints in the Tanimoto chemical similarity coefficient a pair-wise similarity matrix was obtained. The formula for Tanimoto calculations was T (A, B) = AB/(A + B) − AB, where AB are the substructure bits found in both A and B compounds.

### Calculating a chemical similarity tree and its decomposition

The pair-wise similarity matrix was then clustered using hierarchical clustering (hcl) method in R with average linkage parameters. The output tree of the hcl was divided into clusters using the dynamicTreecut^44^ package in R. The tree was further converted into a phylogenetic tree object using the ape package in R and visualized as circular tree plot.

### Enrichment statistics

The compound-term mapping was used as set definitions for set enrichment analysis. We have used the ‘one sample KS test’ to test a null hypothesis that p-values for metabolites in a set are obtained from a reference uniform probability distribution of p-values as defined by the “punif” parameters of R. FDR were calculated using p.adjust function in R for set level p-values.

### Web-application and deployment

A web application was developed in the opencpu R package^45^ to make ChemRICH available to non-R experts. The tool is also available as an R-package. The tool has been deployed at www.chemrich.fiehnlab.ucdavis.edu.

### Source-code availability

The source for the ChemRICH and documentation is available at www.github.com/barupal/chemrich under the cc-by license.

### Statistical analysis

Student *t*-tests were performed in R. Fold changes were calculated by taking the ratio of medians of the two experimental groups in the NOD mouse study. P-values were adjusted using the false discovery rate method in R. Statistical plotting was done using ggplot2 package in R.

## Results

### Metabolome data

**Figure 1:**
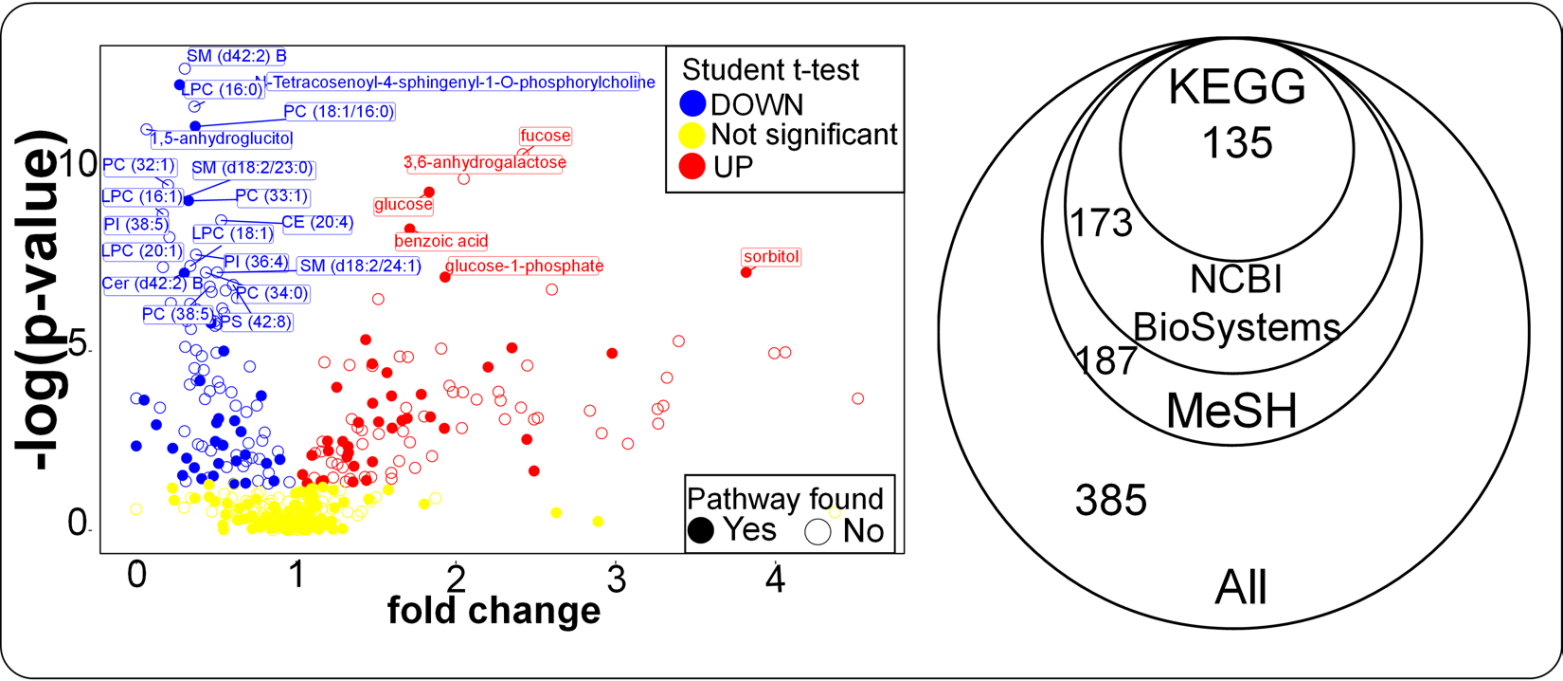
Metabolic dysregulations and its mapping against pathway and ontology databases. *Left:* Volcano plot showing the metabolic dys-regulation in NOD diabetic mice, detailing which of the most significantly altered metabolites were not mapped to metabolic pathways. Right: overlap of all detected metabolites in pathway and chemical ontology databases.

For demonstrating the chemical enrichment clustering and statistics approach in ChemRICH, we used a publicly available plasma metabolomics dataset from a non-obese type 1 diabetes mouse model^23^. Data were downloaded from the NIH MetabolomicsWorkbench.org database^29^ as study number ST000075. Non-obese diabetic (NOD) mice are a polygenic animal model and exhibit a susceptibility to spontaneously develop of type 1 diabetes as an autoimmune result of insulitis. We compared metabolic phenotypes of 36-week old animals that developed diabetes (*n* = 31) versus NOD mice that remained normoglycemic (*n* = 40). In this study, a total of 385 unique plasma metabolites were identified and quantified by normalized signal intensity by three metabolomic assays, untargeted primary metabolism screening by gas chromatography-time of flight mass spectrometry^46^, untargeted profiling of complex lipids by charged surface hybrid liquid chromatography-quadrupole time of flight mass spectrometry^47^, and targeted analysis of oxylipins^48^ by reversed phase liquid chromatography-QTRAP tandem mass spectrometry. Details are provided in Supplement Table S1. More than half of all identified compounds were found to be potentially associated with hyperglycemia using a Student’s t-test (raw *p* < 0.05) with an effect size ranging from 1.05 to 4.5-fold. 22% of all compounds showed a significant 2-fold-change (increased or decreased), highlighting large metabolic differences in diabetic mice beyond glucose metabolism. Figure 1 (left panel) gives an overview on fold-changes and univariate statistical significance in a volcano plot. We found an almost equal number of significantly increased and decreased compounds.

First, we queried all identified metabolites against the NCBI BioSystems pathway repository using their PubChem compound identifiers. This repository covered all major metabolic pathway databases including KEGG^31^, BioCyc^39^, WikiPathways^49^, Reactome^38^ and Gene ontology^50^ therefore it provided most comprehensive pathway annotations for metabolites. We found that 57% of all the known metabolites failed to be annotated to known biochemical pathways in this repository. Specifically, complex lipids and oxylipins lacked pathway annotations (Fig. 1 and Supplement Table S1). We concluded pathway maps failed to provide biochemical overview sets for most identified metabolites in this typical metabolomic study. In addition, many metabolites are mapped to multiple overlapping pathways.

### Development of the ChemRICH approach

**Figure 2:**
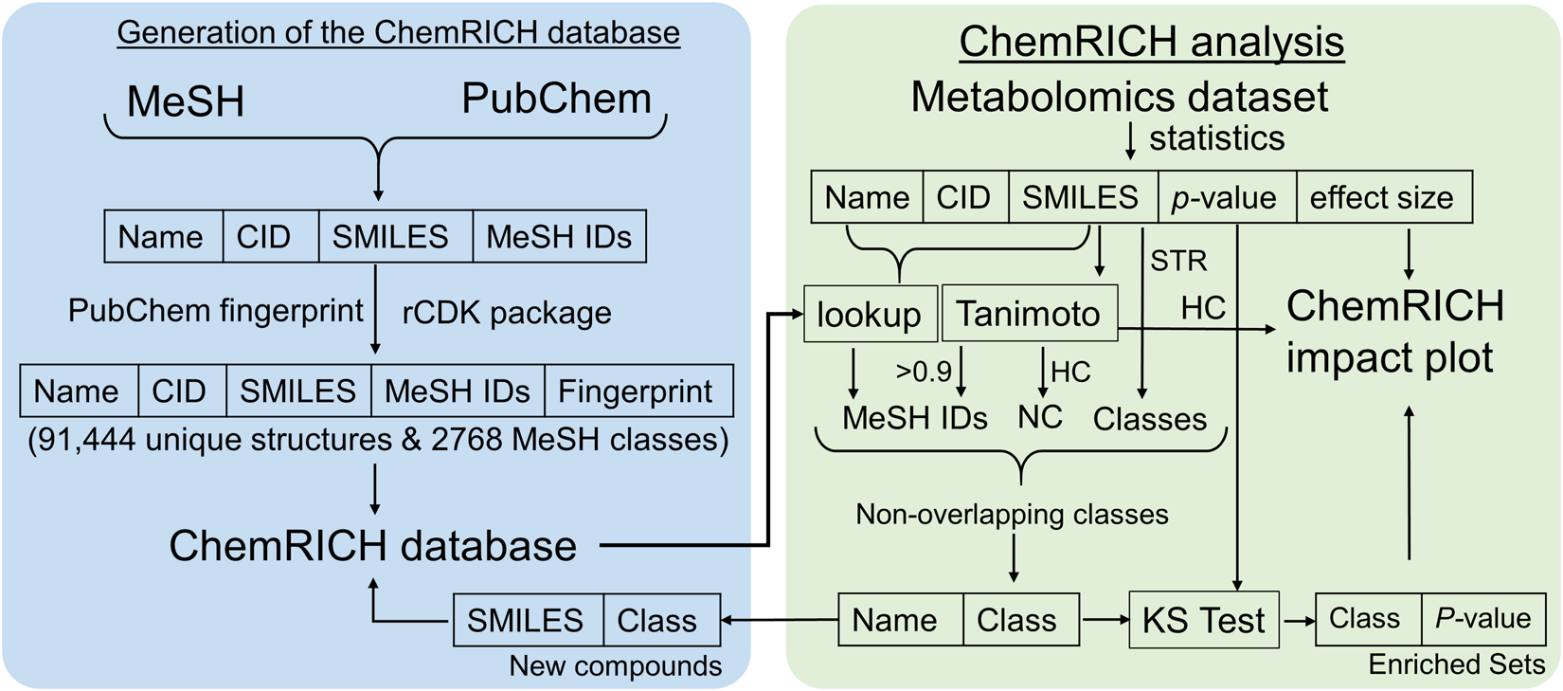
Static and dynamic component of ChemRICH approach. The left panel shows the steps to generate the ChemRICH database using MeSH and PubChem databases and the rCDK package in R. The right panels show the steps in ChemRICH enrichment analysis. It includes finding non-overlapping chemical sets for a list of metabolites from a metabolomics study and then calculating the set level significance using the KS test. Abbreviations: MeSH - Medical Subject Headings, CID - PubChem compound identifiers, SMILES - Simplified molecular-input line-entry system, FA - Fatty acids, NC - New clusters, HC – Hierarchical clustering, STR – string search, CDK - Chemistry Development Kit, KS Test- Kolmogorov–Smirnov test.

**Figure 3:**
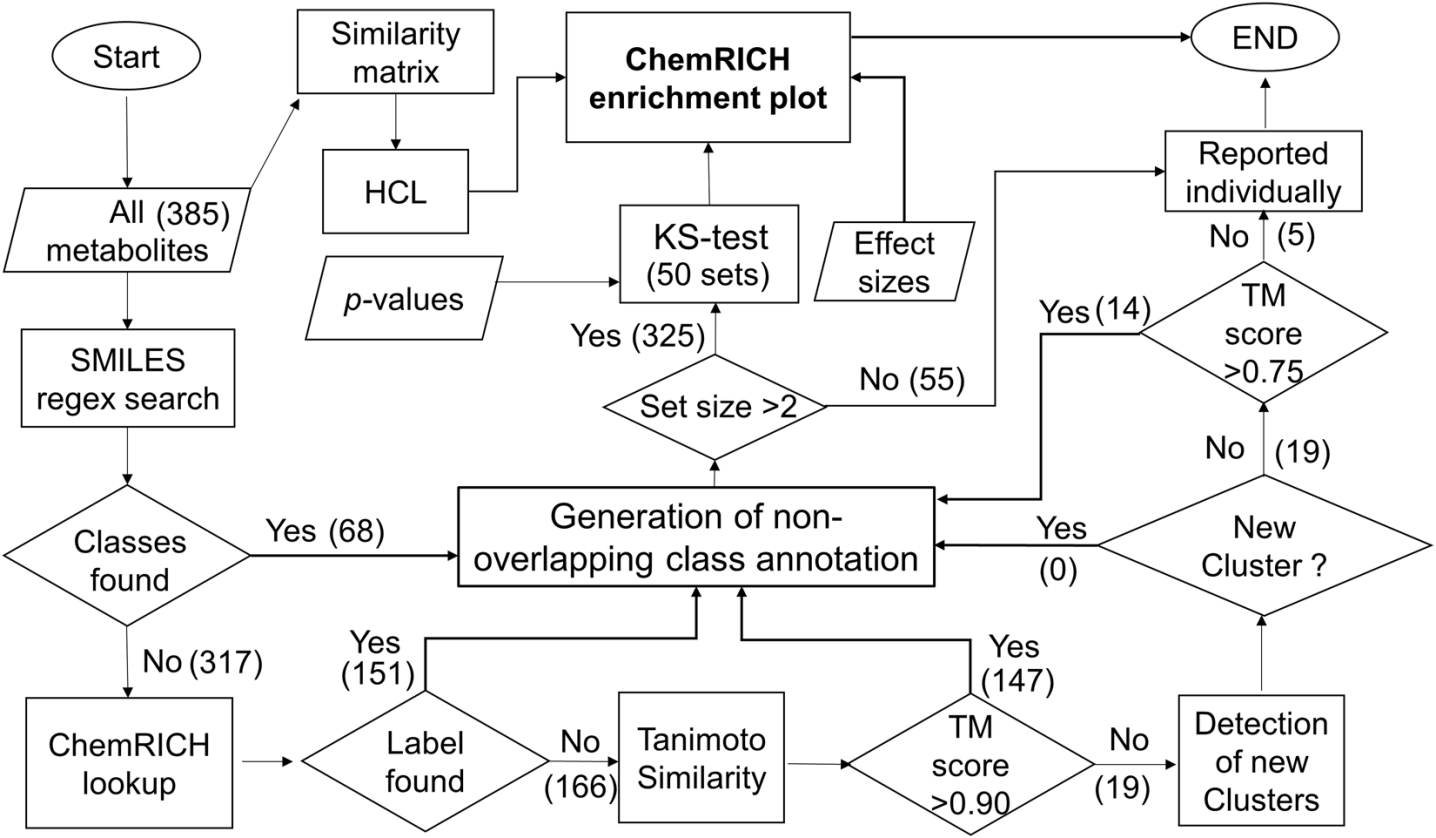
Flowchart of steps in the ChemRICH approach. Number in parentheses are for the test study. Abbreviations: FA – fatty acids, HCL – hierarchical clustering, TM – Tanimoto.

To obtain set definitions for all identified compounds in a metabolome study, we propose to utilize chemical ontology terms and chemical similarity mapping. We named this new approach, ChemRICH. ChemRICH relies on a starting database of metabolites along with their MeSH terms. This starting metabolite list includes all compounds that have been annotated with MeSH terms by the PubChem database. First PubChem CIDs, names and SMILES codes of compounds from the test study were searched in the ChemRICH database and their MeSH terms were retrieved. Next, MeSH terms were estimated using Tanimoto chemical similarity coefficients for the compounds that were not present in the ChemRICH database. MeSH terms for compounds found in newly entered metabolomic studies are now automatically added to the ChemRICH database. The overall development and organization of ChemRICH is shown in the Fig. 2. Precise steps in the use of ChemRICH for the test study are shown in the Fig. 3, including how to define non-overlapping sets of metabolites and how to use the KS-test for obtaining statistical significance for set enrichments. In the following sections we explain the rationale behind using the MeSH ontology, chemical similarity and the KS test for the ChemRICH enrichment analysis.

### Medical subject headings provide metabolite sets defined by chemical classes

We started deriving metabolomic clusters for set enrichments using chemical ontology classes from the MeSH database. Chemical ontologies are useful because they represent a comprehensive and logical approach to classify all detected compounds in a metabolome study. These classes further provide meaningful naming of groups of metabolites, and in some cases, also lend biological relevance. Using the chemical ontology database MeSH, 49% of all identified metabolites of our test NOD mouse metabolome dataset were covered (Fig. 1 and Supplement Table S1). CID, compounds names and SMILES codes were used to query compounds from the MeSH database.

While chemical ontology classification provided better metabolite coverage than biochemical pathway mapping, ontology classes could not directly be used for set enrichment statistical analysis. First and foremost, 51% of the identified compounds were not covered by MeSH. Second, many metabolites were annotated by more than one ontology class. Moreover, many novel metabolites might be detected that have not been annotated in existing databases.

### Chemical similarity improves mapping of metabolite and sets

Novel compounds are frequently found that are not yet present in existing chemical ontologies, for example, triglycerides with unusual odd-chain fatty acids that may enter the blood stream as dietary components. Similarly, compounds with in-silico predicted mass spectra^51^ are often missing from ontology databases. We first calculated Tanimoto similarity coefficients for all unmapped metabolites (51%) using the rCDK package that provides the PubChem 881 bit substructure fingerprint in *R*. These substructure fingerprints were used to calculate Tanimoto similarity coefficients for all query compounds against 91,444 chemicals that are currently included in the MeSH database. All unmapped metabolites were added to the ontology class in which the most similar compounds were found at Tanimoto similarity scores > 0.90. If no ontology class with compounds at Tanimoto score > 0.9 were found, a slightly relaxed cutoff >0.75 Tanimoto score was used. This approach yielded MeSH annotations for up to 98% of total compounds (Fig. 3). Classic fatty acids and their variants, oxylipins, were found to have high Tanimoto similarities. We used a rule based approach to directly establish cluster memberships for each compound. SMILES code string patterns were searched against the SMILES for all the known compounds in the test data set using regular expression analysis in R. With this method, all 14 fatty acids, 51 oxylipins and 3 prostaglandins were correctly assigned to different clusters.

**Figure 4:**
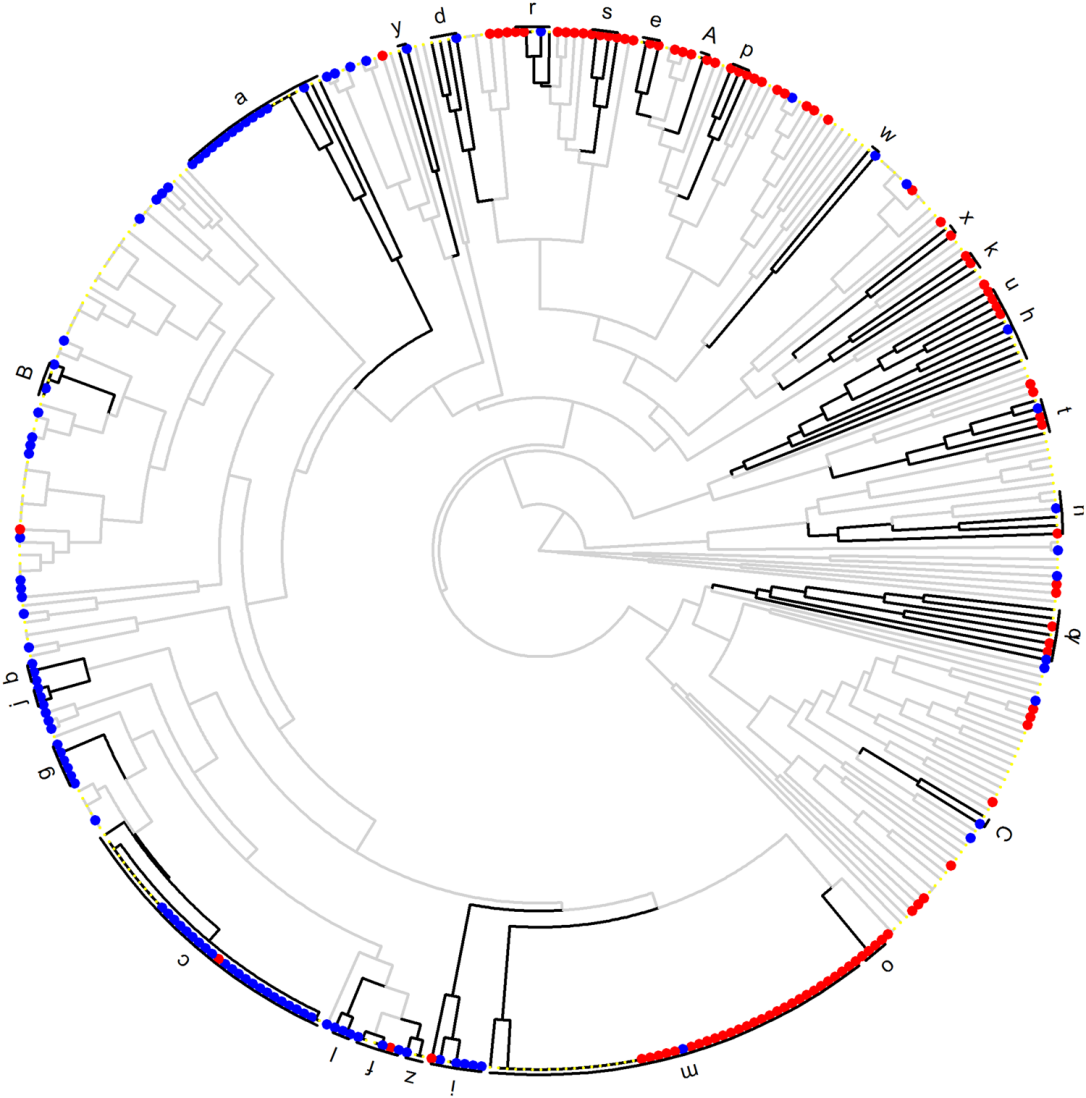
Tanimoto chemical similarity mapping of all identified metabolites in the non-obese diabetic mouse dataset. Clusters are defined by comparing within- versus between group similarities, forming a clustered chemical similarity tree. Dark black lines indicate boundaries of clusters that are significantly different in diabetic versus non-diabetic NOD mice (*p* < 0.05). Cluster letter labels are detailed in Supplement Table S1. Increased metabolites levels in diabetic mice are labeled as red nodes, decreased levels are marked in blue.

We also tested the idea of using chemical similarity alone to generate sets of chemicals that could be used for calculating enrichment statistics. The cluster detection algorithm DynamicTreeCut^44^ had been developed for cluster detections in genomic data. We found that the algorithm also efficiently distinguishes groups of chemicals if the average Tanimoto score within a set is significantly higher than the scores between a cluster and all other clusters. If only chemical similarity is used this process yielded a list of 89 clusters that generally could be used to highlight sets of metabolites that are significantly different to a control group in a metabolomic study. Such a chemical similarity clustering tree is shown in Fig. 4, with cluster labels given in Supplement Table S1. However, pure chemical similarity clusters are difficult to name by intelligible, biologically or chemically meaningful class labels.

Instead, we used Tanimoto chemical similarity mapping in addition to the backbone of MeSH ontology. Tanimoto chemical similarity calculations enabled us to generate new clusters that were absent in the MeSH database. New clusters of chemicals might be produced whenever a completely new class of metabolites is discovered by metabolomics, for example, the fatty acyl esters of hydroxyl fatty acids (FAHFAs)^52,53^. In our given NOD mice data set, no FAHFA lipids were detected. Instead, we tested the concept by excluding the ‘*saturated sphingomyelins*’ chemical ontology definition from the list of known distinct sets. Indeed, we found that the actual saturated sphingomyelins in the NOD mice metabolomics dataset were identified as a distinct and novel set by the chemical similarity tree cutting approach^44^. This test showed that not only can Tanimoto chemical similarity add novel metabolites to the tree that lack MeSH chemical ontology information, but the dynamic tree cut approach automatically detects entire new groups of compounds and adds these compounds into the ChemRICH sets as a new cluster for set enrichment statistics. Final MeSH annotations for each metabolite are provided in Supplemental Table S1.

### Defining non-overlapping ontology classes for metabolites

By combining chemical similarity and MeSH ontology mappings we obtained non-overlapping classes for a maximum number of structurally identified metabolites. These classes formed the metabolite sets that were subsequently used for calculating set enrichment statistics. Non-overlapping classes overcome two major bottlenecks of set enrichment analyses. In non-overlapping sets each metabolite is used only once, avoiding bias for hub metabolites such as glutamate that is used in many different biochemical reactions. Second, non-overlapping sets avoid the need for correction for multiple hypothesis testing for sets with high overlaps.

Naming these sets is performed in the following way in ChemRICH: first, all superclass and subclass annotations are retrieved for each compound and sorted by specificity as defined by the MeSH ontology tree structure. For instance, the metabolite ‘*betaine*’ maps to the class entry D02.675 as ‘*onium compounds’* but also to the more specific child term D02.092.877 ‘*quaternary ammonium compounds*’. Compounds were associated to their most specific ontology classes until a minimum of three compounds per class was reached. This approach found 49 non-overlapping MeSH ontology classes for 85% of the identified metabolites in our NOD dataset. We named these classes ChemRICH sets. The residual number of 15% of the identified compounds remained annotated with their individual MeSH ontology.

### ChemRICH sets enabled self-contained enrichment analysis of altered metabolites

The combined number of metabolites in ChemRICH sets were then defined as study-specific, background-independent database to be used for the calculation of metabolite-set enrichment *p*-values.

There are two statistical tests that can be used for statistical set enrichment that do not rely on background databases, the binomial test and the Kolmogorov–Smirnov (KS) test. Binomial tests require a *p*-value cutoff to define the significantly affected variables within a set, while the KS-test does not require such a threshold and is therefore more comprehensively including all metabolites in a metabolomic data set. The KS test obtains set-level significance *p*-values by comparing the cluster *p*-values against a theoretical *p*-value distribution (“puinf” in *R*). Hence, the KS test yields set level *p*-values that are not affected by any (biochemical) background database or by the total number of altered metabolites, unlike the commonly use hypergeometric pathway enrichment test. Moreover, the KS-test also included compounds with marginal p-values, unlike the bionomial enrichment statistics test.

**Figure 5:**
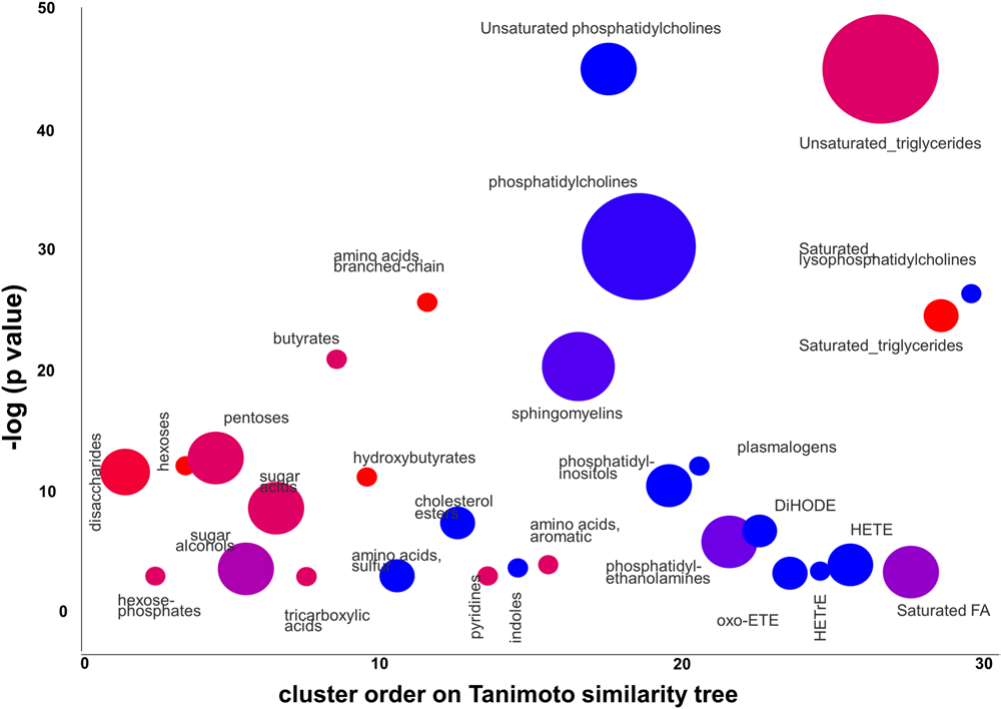
ChemRICH set enrichment statistics plot. Each node reflects a significantly altered cluster of metabolites. Enrichment *p*-values are given by the Kolmogorov–Smirnov-test. Node sizes represent the total number of metabolites in each cluster set. The node color scale shows the proportion of increased (red) or decreased (blue) compounds in diabetic NOD mice compared to control mice. Purple-color nodes have both increased and decreased metabolites.

We compared all identified NOD mouse plasma metabolites for diabetic versus non-diabetic mice using the ChemRICH sets, using the Kolmogorov–Smirnov-test for set enrichment statistical analysis. Results are visualized in a 2-dimensional scatter plot (Fig. 5) in which sets were sorted by their order on the chemical similarity tree (Fig. 4), a graph that is intentionally similar to pathway mapping graphs obtained from MetaboAnalyst^27^. This graph directly visualizes the most significant and largest metabolite sets organized according by overall chemical diversity across all identified metabolites. Enrichment results for individual cluster sets are given in Table 1. We here show for the first time a chemical ontology-based metabolite set definition in combination with chemical similarity calculations for calculation of metabolic set enrichment significance.

**Table 1:** Results of the ChemRICH enrichment analysis for the altered metabolites in the NOD mice study. Abbreviations - PC: Phosphatidylcholines, LPC: Lysophosphatidylcholines, BCAA: Branched chains amino acids, TG: Triacylglycerols, PI: Phosphatidylinositol, PE: Phosphatidylethanolamine.

Cluster name	Cluster size	p-values	FDR	Counts of altered	Increased	Decreased
Unsaturated_LPC	9	2.2E-20	1.1E-18	9	0	9
Unsaturated_TG	46	1.5E-15	3.7E-14	31	30	1
PC	44	5E-13	8.2E-12	29	1	28
Saturated_LPC	3	2.7E-12	3.4E-11	3	0	3
BCAA	3	5.8E-12	5.7E-11	3	3	0
Saturated_TG	4	1.6E-11	1.3E-10	4	4	0
butyrates	3	6.1E-10	4.3E-09	3	2	1
sphingomyelins	16	1.1E-09	6.8E-09	13	1	12
hexoses	9	0.0000022	0.000012	7	6	1
pentoses	3	0.0000041	0.000018	3	3	0
plasmalogens	3	0.0000041	0.000018	3	0	3
disaccharides	7	0.0000071	0.000029	6	6	0
hydroxybutyrates	3	0.000011	0.00004	3	3	0
PI	6	0.000022	0.000076	5	0	5
sugar acids	9	0.00014	0.00046	6	6	0
cholesterol esters	4	0.00051	0.0016	3	0	3
DiHODE	4	0.001	0.003	2	0	2
PE	9	0.0023	0.0063	5	1	4
indoles	3	0.016	0.04	2	2	0
amino acids, aromatic	3	0.02	0.048	2	0	2
sugar alcohols	9	0.022	0.051	4	3	1
Saturated FA	9	0.027	0.057	3	2	1
HETrE	3	0.027	0.057	2	0	2
HETE	6	0.028	0.057	3	0	3
oxo-ETE	4	0.03	0.058	2	0	2
amino acids, sulfur	4	0.038	0.07	2	0	2
pyridines	3	0.039	0.07	2	2	0
hexosephosphates	3	0.04	0.07	2	2	0
tricarboxylic acids	3	0.041	0.07	2	2	0

### ChemRICH enrichment analysis improves biological and biochemical interpretations

This dataset on non-obese diabetic versus normoglycemic mice has been published and carefully interpreted previously^23^. The authors noted a range of overt changes such as ‘elevations in circulating triacylglyercides’ and ‘reductions in major structural lipids, most notably lysophosphatidylcholines and phosphatidylcholines’^23^. However, the authors noted only a total 18 manually annotated clusters of identified metabolites^23^ whereas ChemRICH refined the annotation of systematically different metabolite clusters into 55 distinct sets. The authors visualized the data in simple Tanimoto chemical similarity maps^40,54^ that obscured a very important difference in the diabetic and non-diabetic mice: all complex lipids were quite distinctly regulated between lipids with unsaturated fatty acyl groups, and complex lipids that consisted of only saturated fatty acyl chains^54^. Both cluster sizes and significance values for unsaturated triglycerides, unsaturated phosphatidylcholines, unsaturated sphingomyelins and a range of other lipid classes were markedly different from their saturated counterparts (Fig. 5 and Table 1). Similarly, ChemRICH enrichment analysis very clearly points out the high significance of increased branch-chain amino acids in comparison to the aromatic- or sulfur-containing amino acid sets. Manual classifications as well as univariate statistic interpretations by the original authors did not point out these large differences. Similarly, our ChemRICH statistics showed clear significance differences in carbohydrate metabolism detailed with pentoses, hexoses, disaccharides and sugar acids on the one hand, and much lower significance for hexose phosphates and sugar alcohols on the other hand (Fig. 5). These overt phenotypes were not clearly delineated in the original publication^23^. Detailed carbohydrate interpretations are necessary as it has been reported that endogenous sugar acids may modulate the feeding behaviour of rats^55^ and can also disturb leptin responsive signalling pathway^56^. Without ChemRICH, those refined clusters were not identified and interpretations focus mostly on broad categories such as carbohydrates or lipids as reported in the original paper^23^. Hence, ChemRICH calculations and visualizations improve the ability to perform biological interpretations. Results of ChemRICH analysis for the example NOD mice study are provided in Supplementary Table S1 and S2. ChemRICH has also been tested on a smaller dataset of 128 known compounds^57^ for finding interpretable metabolic clusters (Supplementary Tables S3 and S4).

### ChemRICH is available both online and as stand-alone *R* package

To make ChemRICH broadly available for the metabolomic community, we have used the OpenCPU web-framework to call our *R*-functions via a representational state transfer- application programming interface (REST-API). The input file must include SMILES codes and PubChem compound identifiers for all identified metabolites. This information can be easily obtained from the Chemical Translation Service^58^ and the PubChem identifiers exchange service^59^. ChemRICH is hosted at http://chemrich.fiehnlab.ucdavis.edu. For the bioinformatics community we have coded the tool as *R*-package.

## Discussion

Because of the limitations of pathways maps for the metabolomics data interpretation, we have developed ChemRICH as a next generation of set enrichment analysis tools for metabolomics. ChemRICH is an alternative approach to the pathway analysis. It uses structurally defined metabolite sets and self-contained statistical analysis for finding enriched metabolite sets. It fills a major gap in the interpretation of the metabolomics datasets. Combining chemical similarity and classification ontologies enables annotating and naming metabolites as non-overlapping chemical classes. A self-contained KS test allows discovering metabolite sets with the most statistical significance. The tool has been developed into an R-package and a web-app for straightforward use by the metabolomics community. We demonstrated that ChemRICH can outperform classic tools for visualizing and interpreting metabolomics datasets using a publicly available study and highlighting the chemical sets which were not discussed in the original paper.

Using classic pathway maps covered only 40% of the plasma metabolites identified in our NOD mouse test case. Such low coverage is particularly critical whenever multiple analytical assays are combined to detect metabolites from different chemical classes, including complex lipids or oxylipins. Limiting enrichment analysis to pathway maps underutilizes those datasets. In contrast, combining chemical similarity with established chemical ontologies can classify over 90% of the identified metabolites for enrichment analysis. We have used MeSH ontology classes because these classes are already mapped to the PubMed literature database. This will allow straightforward use of metabolite set labels for interpreting metabolic dysregulation in the context of biochemical enzymes, physiology, chemical exposure or anatomical changes by manual or automated text mining.

ChemRICH is particularly useful for clinical and epidemiological studies using blood or urine specimens. Non-targeted blood metabolomics detects compounds that originate from cellular metabolism as well as exposure compounds of xenobiotic origins including food polyphenols^60^ or drug compounds^13^. ChemRICH efficiently places such exposome chemicals into metabolites sets to perform exposure enrichment analysis that exceeds classic pathway enrichments.

Statistical significance of metabolomics enrichment should not depend on the size of background databases used. Using non-overlapping sets of variables have been used for partitioning and trimming of gene ontology databases^61,62^ as basis for enrichment statistics. Moreover, the KS-test based gene set enrichment analysis (GSEA)^42^ has been widely accepted as the standard approach to perform set enrichment analysis for genomics studies^63–65^. It does not require splitting gene lists, it can take entire genomics dataset as input and it does not depend on any specific background database. We propose metabolomic enrichment tools should learn from these experiences and also use the KS-test as standard for enrichment analysis.

Using chemical similarity instead of fixed definitions of biochemical pathway has several advantages. Using encoded chemical structures allows precise mapping of metabolite to set definitions, avoiding manual, often arbitrary annotations of metabolites to ‘sets’. Importantly, most complex lipids are not mapped to biochemical pathways. Chemical similarity mapping detects classes of compounds that are not yet represented in chemical ontologies, for example, for in-silico predicted compounds by tools such as LipidBlast^51^ or the Metabolome-in-silico-network expansion database, MINE^66^. Adding unmapped metabolites to ontology class by Tanimoto chemical similarity is analogous to predicting ‘anatomical therapeutic chemical code’ for drug-like compounds^67^.

Chemically similar compounds are in biochemical proximity^40^. This provides a strong rationale for chemical clustering of known metabolites and perform set enrichment using the detected clusters. ChemRICH complements MetaMapp which is used for visualizing the chemical diversity of metabolomics datasets using network graphs. Use of MetaMapp and ChemRICH will provide the complementary visualization and summarization outputs that can streamline the interpretation of a metabolomics study. However, the use of ChemRICH is limited by our ability to correctly identify compounds in non-targeted metabolite screens. Future expansions might map unknown metabolites as well using mass spectral similarity or sub-structure prediction from mass spectra. In MetaMapp, we have implemented using mass spectral similarity networks for visualizing large volume of spectral data^68^, including unknown metabolites. Clusters can be detected using a community detection algorithm, but annotating such clusters with clear chemical names, or using these for biological interpretations, is still limited.

## Conclusion

ChemRICH outperforms classical pathway overrepresentation analysis approach for the interpretation of the metabolomics datasets. The approach can be used in studies to uncover biological mechanisms in organisms under a genetic or environmental stress in a system biology manner or finding risk factors for chronic diseases in exposome-wise association studies using blood specimens. ChemRICH is available via http://chemrich.fiehnlab.ucdavis.edu. The source code is available at www.github.com/barupal/chemrich.

## Data availability

Metabolomics dataset is available at the Metabolomics Workbench repository (www.metabolomicsworkbenchlorg) with the accession number ST000075. ChemRICH R package and the code is available at www.github.com/barupal/chemrich

## Electronic supplementary material

Dataset S1-S4
